# Supplementary material for: Perception and memory have distinct spatial tuning properties in human visual cortex
Source: Nat Commun. 2022 Oct 18;13:5864. doi: 10.1038/s41467-022-33161-8 (PMC9579130; doi:10.1038/s41467-022-33161-8)
Supplement: Supplementary file 1 — Supplementary Information [file 41467_2022_33161_MOESM1_ESM.pdf]

Supplementary Figures

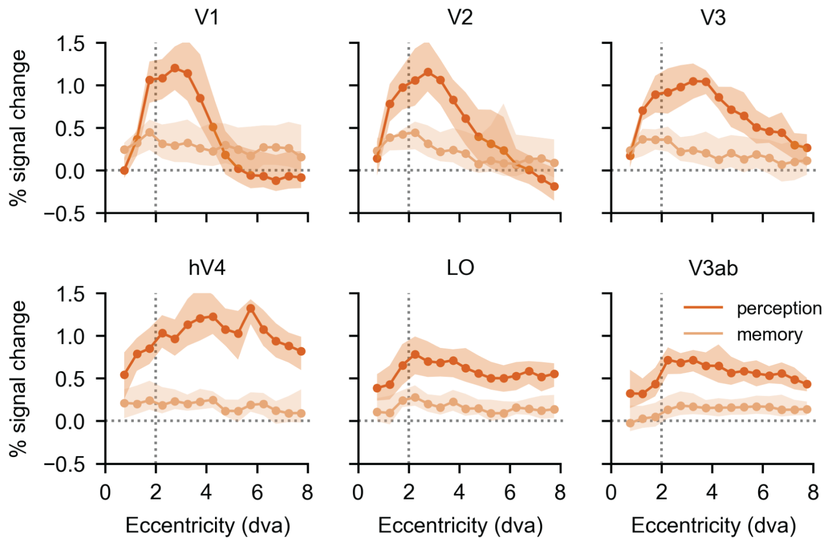

Supplementary Figure 1. Eccentricity response functions. Eccentricity response functions, averaged across all participants and stimuli, are plotted separately for perception and memory. Dots represent the mean BOLD % signal change at different eccentricities. The lines connecting dots interpolate between adjacent points. Shading represents the 95% confidence interval around each point, with linear interpolation between points. The vertical dashed line represents the eccentricity that stimuli were presented at.

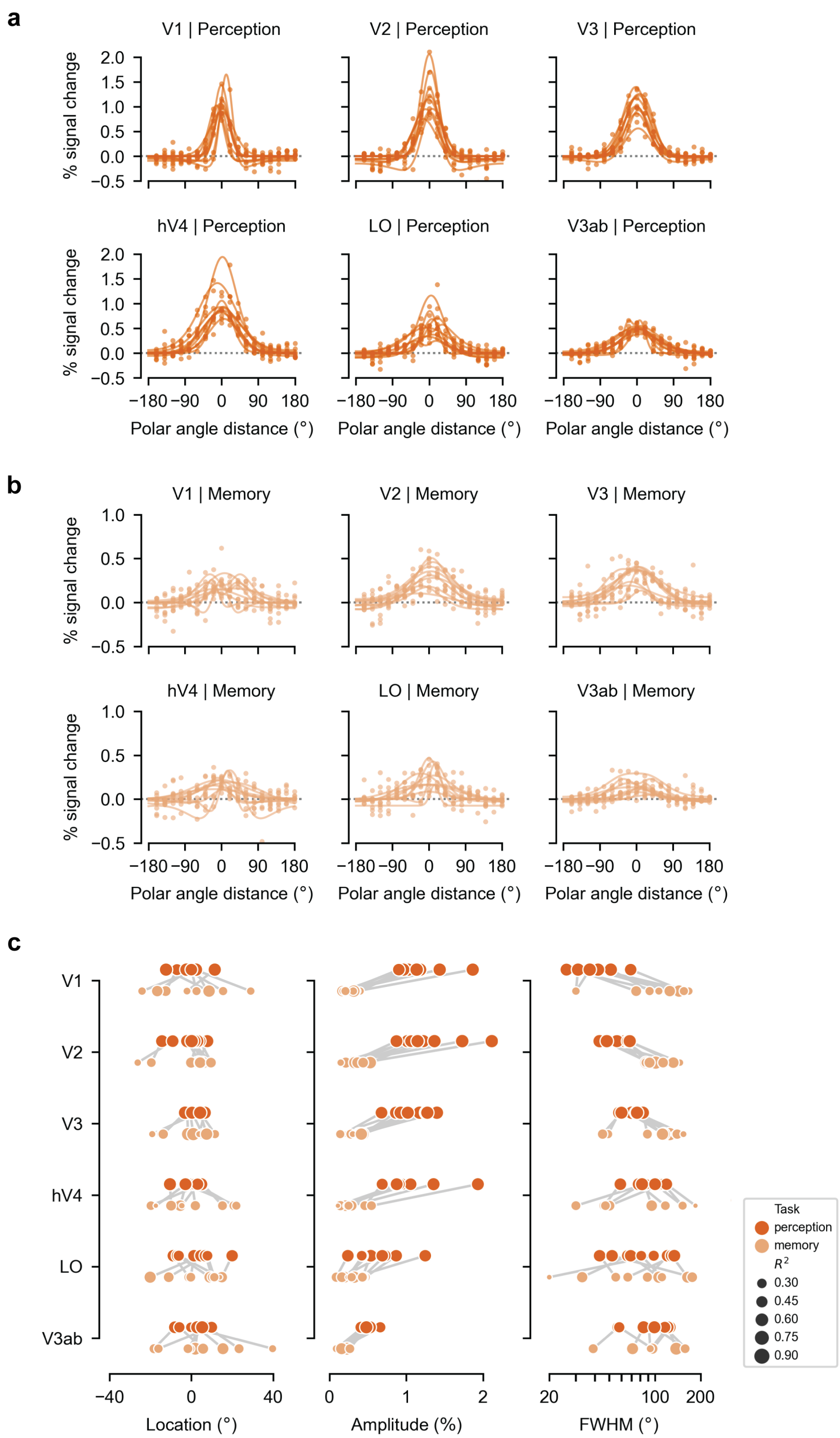

Supplementary Figure 2. Perception and memory responses in individual participants. (a) Perception polar angle response functions are plotted for each of nine participants. Variability in baseline BOLD responses has been eliminated by subtracting the mean offset from 0% signal change in polar angles bins near 180° from all the bins. Lines represent the fit of the difference of two von Mises distributions to each participant's data. **(b)** Memory polar angle response functions and fitted von Mises are plotted for each of nine participants. Methods and conventions as in a. **(c)** Location, amplitude, and FWHM parameters for perception and memory are plotted for each participant. The size of the dots is scaled by the goodness of fit (R^2) of the von Mises fit to that participant's data. Lines join parameters from the same participant.

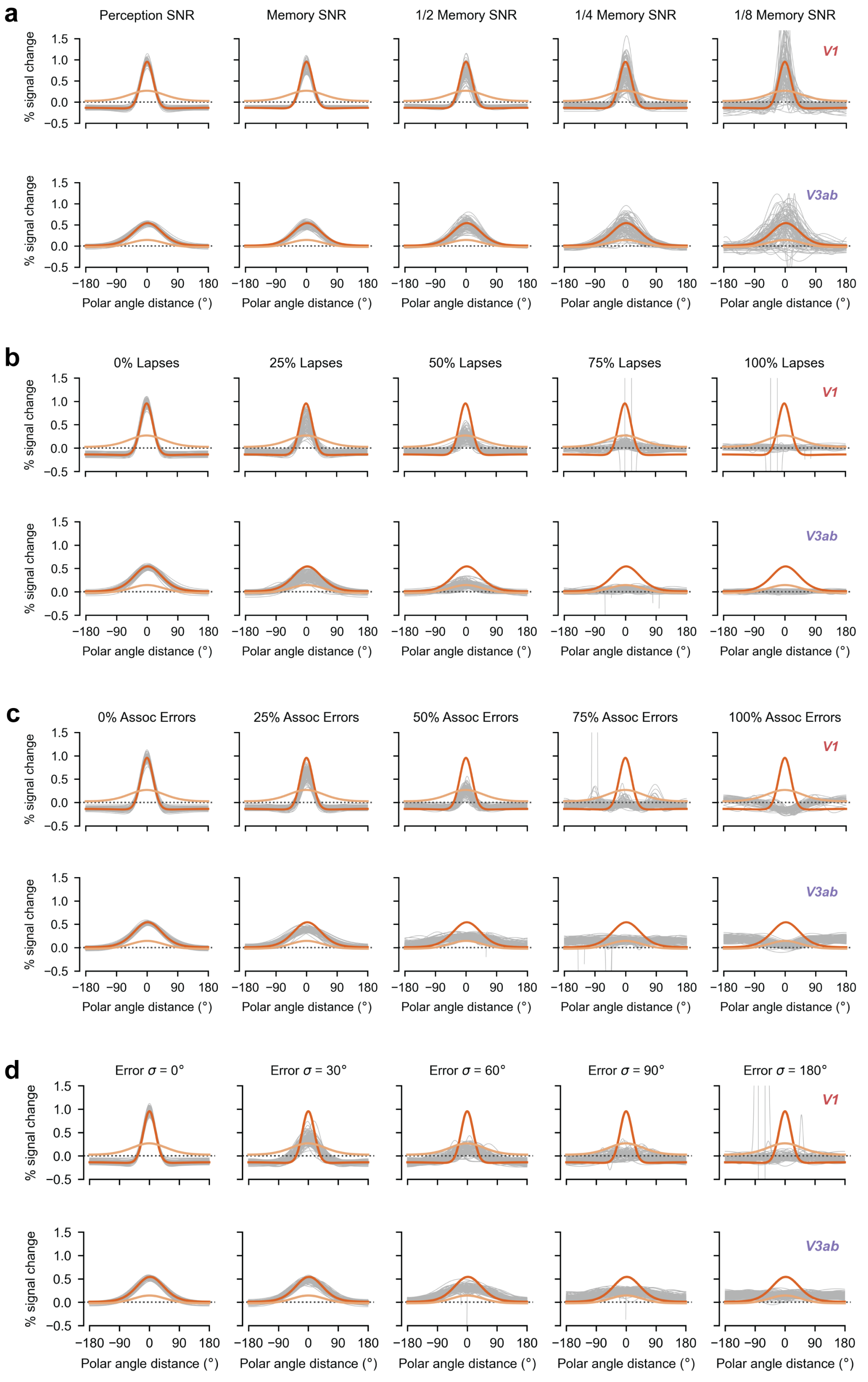

Supplementary Figure 3. von Mises fits to simulated V1 and V3ab datasets with different noise levels. (a) Gray lines represent the von Mises fits to simulated V1 and V3ab perception datasets with different levels of SNR. Each panel contains 100 independently simulated datasets with the same noise level. Orange lines represent the fits to the actual perception and memory data, reproduced from Figure 4b, and are the same for each SNR value within an ROI. **(b)** Gray lines represent the fits to simulated V1 and V3ab perception datasets with different frequencies of retrieval task lapses. Other conventions as in a. **(c)** Gray lines represent the fits to simulated V1 and V3ab perception datasets with different frequencies of associative memory errors. Other conventions as in a. **(d)** Gray lines represent the fits to simulated V1 and V3ab perception datasets with different amounts of angular memory error. Other conventions as in a.

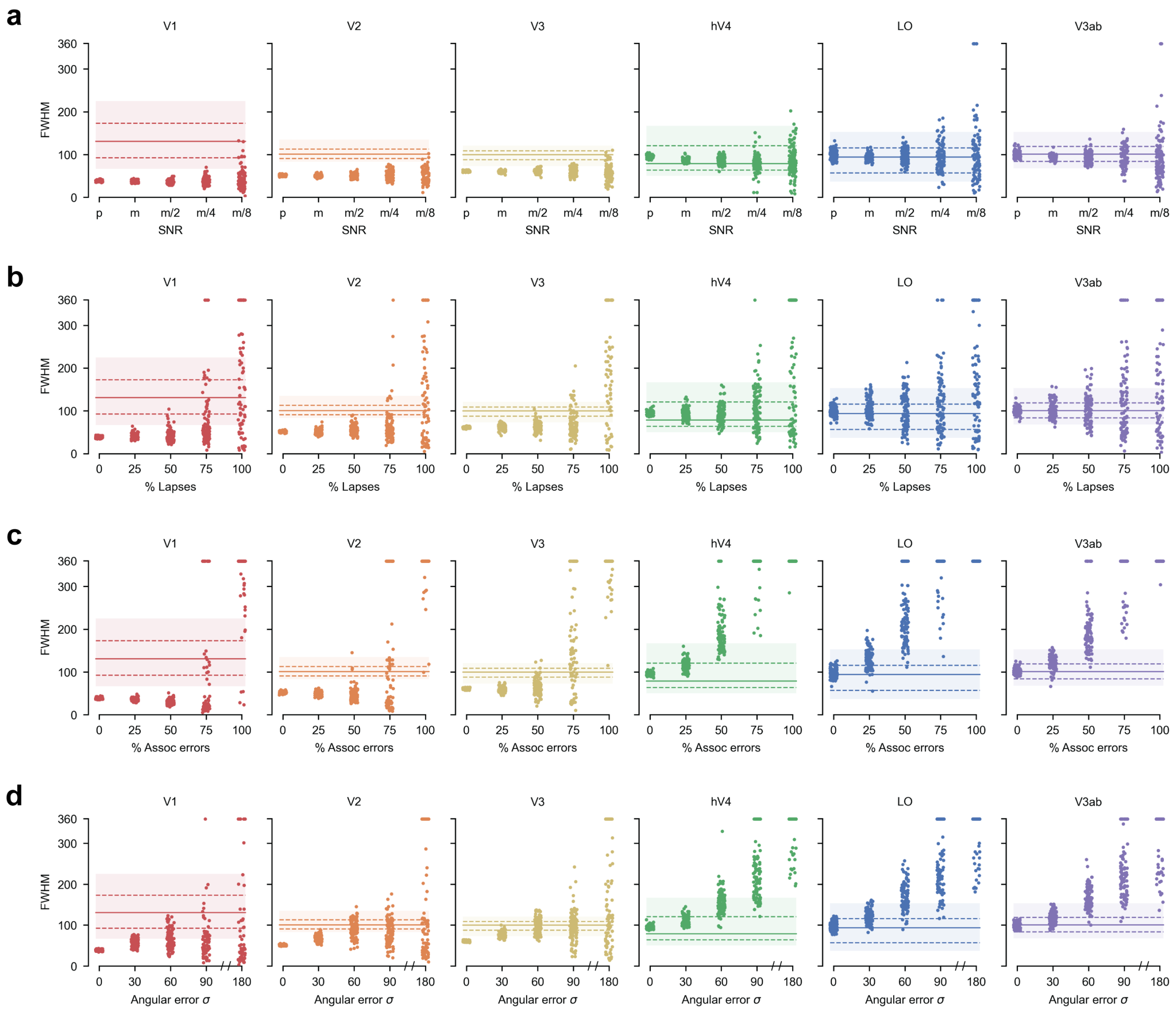

Supplementary Figure 4. FWHM parameters for simulated datasets with different noise levels. (a) Colored dots represent the FWHM parameters from simulations with different levels of SNR. Each x position within a panel contains parameters from 100 independently simulated datasets with the same noise level. Solid horizontal lines represent the memory FWHM for that ROI, reproduced from Figure 4c. Dashed lines represent the 68% confidence interval over that value and shaded area represents the 95% confidence interval, reproduced from Figure 4c. Note that these data are plotted on a linear scale but the data in 4c are plotted on a log scale. Fewer than 100 visible dots should be interpreted as clustering at FWHM = 360° or failure to successfully fit a difference of von Mises. **(b)** Colored dots represent the FWHM parameters from simulations with different frequencies of retrieval task lapses. Other conventions as in a. **(c)** Colored dots represent the FWHM parameters from simulations with different frequencies of associative memory errors. Other conventions as in a. **(d)** Colored dots represent the FWHM parameters from simulations with different amounts of angular memory error. Other conventions as in a. See Figure 5 for the percentage of dots falling within the 95% confidence intervals.

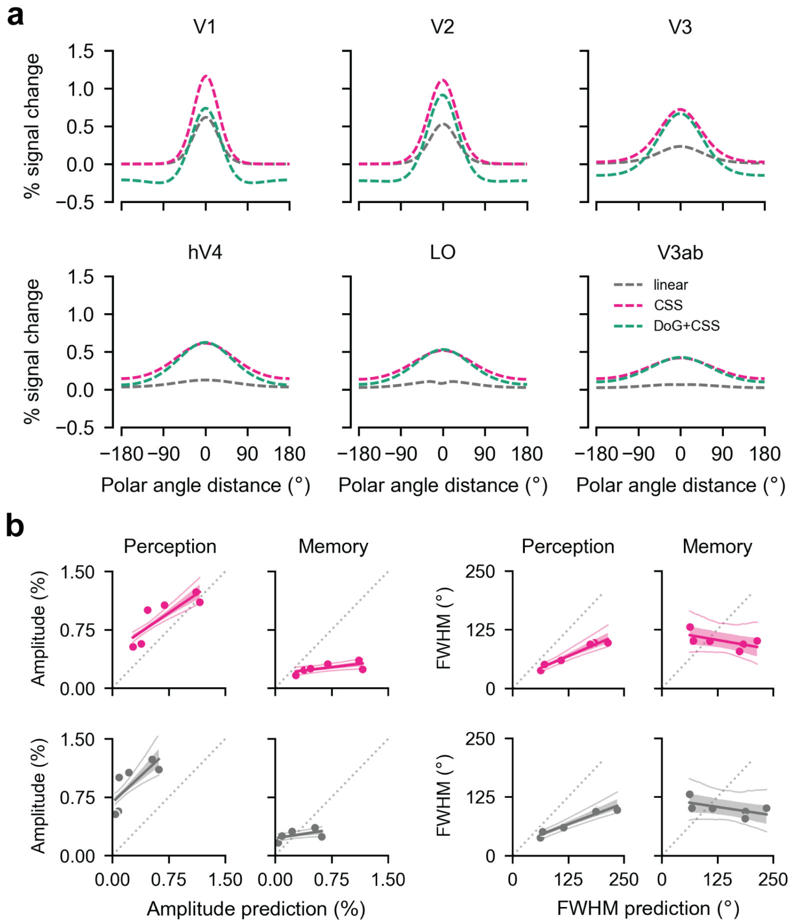

Supplementary Figure 5. pRF model comparisons. (a) Predicted polar angle response functions are plotted for three pRF models: linear, CSS, and DoG+CSS. Comparing these predicted responses to perception data plotted in Figure 4b, the linear model did a poor job of predicting perception responses in later visual areas, while the other two models did well in all areas. The DoG+CSS model selectively captured negative responses in V1–V3. Comparing the predicted responses to memory data plotted in Figure 4b, all models did a poor job of predicting memory responses in early visual areas. **(b)** Predicted versus observed amplitude (left) and FWHM (right) are plotted separately for perception and memory and for the CSS (pink) and linear (gray) pRF models. Each dot represents an ROI. Lines represent the line of best fit across the dots. The shaded regions are the bootstrapped 68% confidence intervals generated from resampling participants with replacement, and the thin lines indicate the bootstrapped 95% confidence intervals. See Figure 6 for DoG+CSS model.

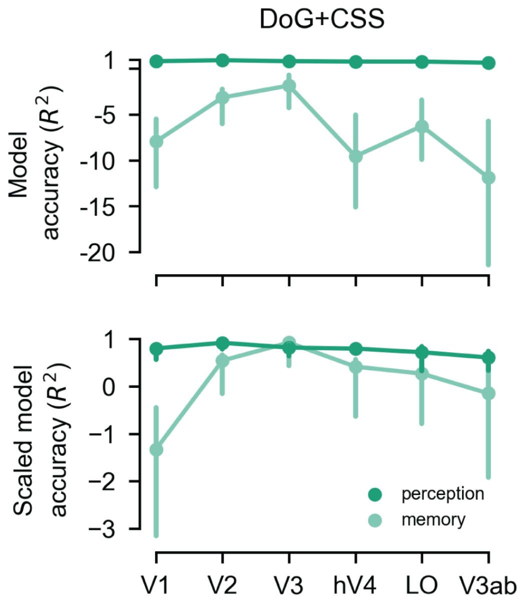

Supplementary Figure 6. pRF model goodness-of-fit. Top: Model accuracy (R^2) of the predicted polar angle response functions for the DoG+CSS pRF model, evaluated separately for perception and memory data from each ROI. Dots indicate R^2 and error bars indicate bootstrapped 68% confidence intervals. R^2 is worse for memory than perception in every ROI by large margins. Bottom: Model accuracy (R^2) of the predicted polar angle response functions after separately rescaling the pRF predictions to best fit the perception and memory data. Memory R^2 is improved after rescaling but still inferior to perception R^2, especially in V1.

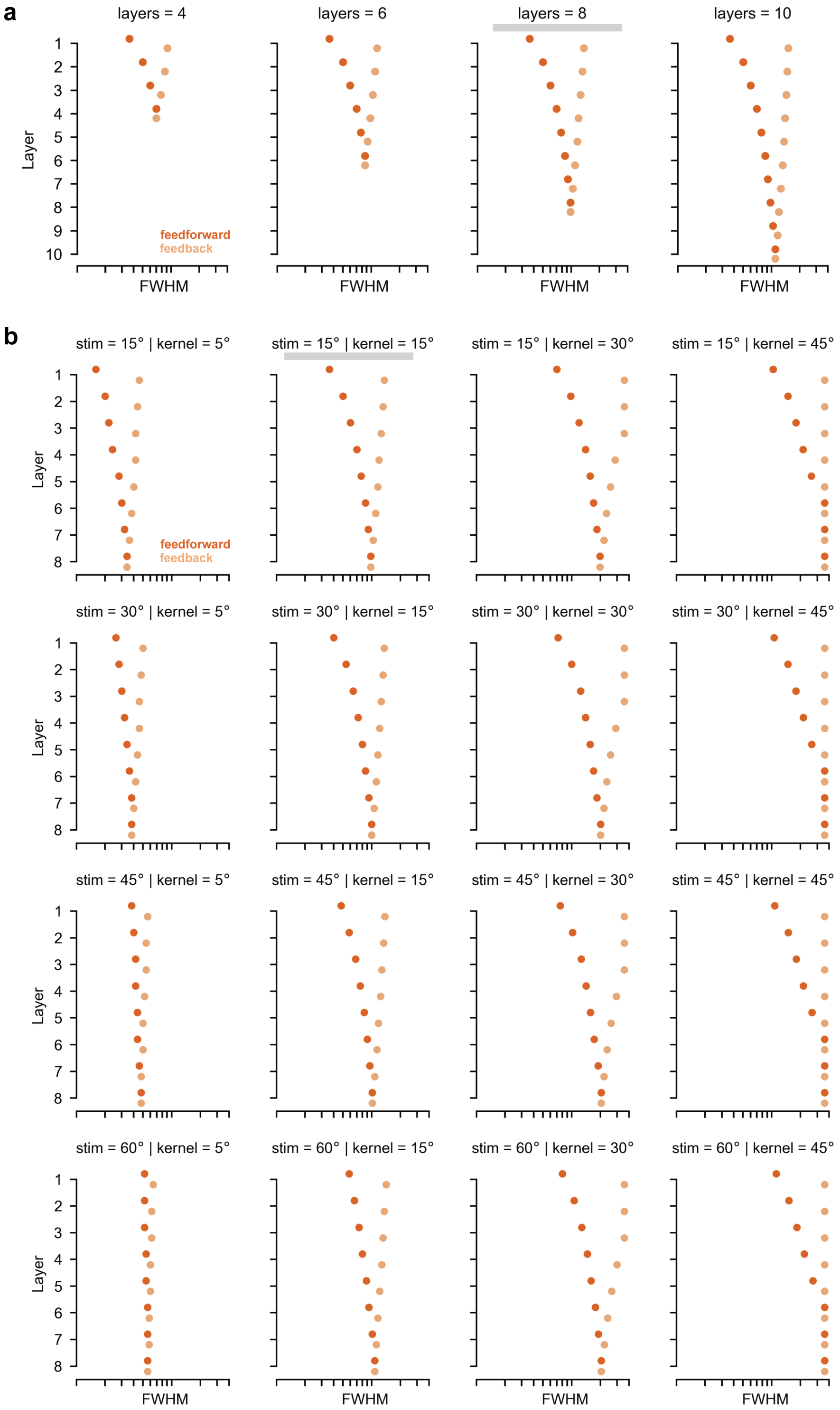

Supplementary Figure 7. Impact of parameter choices on the FWHM of hierarchical network model activity. (a) Impact of the number of layers on feedforward and feedback simulations. A model with fixed stimulus and kernel parameters (stimulus = 15°; kernel σ = 15°) was run with 4, 6, 8, and 10 layers. While the overall pattern does not change across layers, the exact values in a given layer depend on the the total number of layers. For instance, note the difference between perception and memory FWHM in layer 4 in the 4 layer model versus the 10 layer model. The gray bar indicates the exact model that appears in Figure 7c. **(b)** Impact of stimulus size and kernel size on feedforward and feedback simulations. An 8 layer model was run with variable stimulus size (rows) and kernel size (columns). Stimulus size and kernel size interact to determine the amount of change in FWHM across layers. For each stimulus size, larger kernel sizes produce larger changes in FWHM (compare left vs middle columns). However, when the kernel is very large, FWHM saturates quickly (right column). Given small kernel sizes, large stimuli produce higher starting values of FWHM but smaller changes in FWHM across layers than small stimuli (compare bottom left to top left).
