# Supplementary material for: Perception and memory have distinct spatial tuning properties in human visual cortex
Source: Nat Commun. 2022 Oct 18;13:5864. doi: 10.1038/s41467-022-33161-8 (PMC9579130; doi:10.1038/s41467-022-33161-8)
Supplement: Supplementary file 2 — Reporting Summary [file 41467_2022_33161_MOESM2_ESM.pdf]

nature portfolio

Corresponding author(s): Serra Favila

Last updated by author(s): Sep 1, 2022

Reporting Summary

Nature Portfolio wishes to improve the reproducibility of the work that we publish. This form provides structure for consistency and transparency in reporting. For further information on Nature Portfolio policies, see our Editorial Policies and the Editorial Policy Checklist.

Statistics

For all statistical analyses, confirm that the following items are present in the figure legend, table legend, main text, or Methods section.

n/a	Confirmed	
☐	☒	The exact sample size (*n*) for each experimental group/condition, given as a discrete number and unit of measurement
☐	☒	A statement on whether measurements were taken from distinct samples or whether the same sample was measured repeatedly
☐	☒	The statistical test(s) used AND whether they are one- or two-sided<br>*Only common tests should be described solely by name; describe more complex techniques in the Methods section.*
☒	☐	A description of all covariates tested
☐	☒	A description of any assumptions or corrections, such as tests of normality and adjustment for multiple comparisons
☐	☒	A full description of the statistical parameters including central tendency (e.g. means) or other basic estimates (e.g. regression coefficient) AND variation (e.g. standard deviation) or associated estimates of uncertainty (e.g. confidence intervals)
☐	☒	For null hypothesis testing, the test statistic (e.g. *F*, *t*, *r*) with confidence intervals, effect sizes, degrees of freedom and *P* value noted<br>*Give P values as exact values whenever suitable.*
☒	☐	For Bayesian analysis, information on the choice of priors and Markov chain Monte Carlo settings
☒	☐	For hierarchical and complex designs, identification of the appropriate level for tests and full reporting of outcomes
☒	☐	Estimates of effect sizes (e.g. Cohen's *d*, Pearson's *r*), indicating how they were calculated

Our web collection on statistics for biologists contains articles on many of the points above.

Software and code

Policy information about availability of computer code

Data collection	Data were collected using PsychoPy 1.85.6
Data analysis	fMRI data were preprocessed using FSL 5.0.10, reesurfer 5.3.0, and nipype 1.1.9. Population receptive field models were fit using Vistasoft 1.0. GLMs were fit using GLMdenoise 1.4. Data were quantified and visualized using nibabel 3.2.1, numpy 1.21.2, scipy 1.7.1, pandas 1.3.3, matplotlib 3.4.3, and seaborn 0.11.2. Analysis code required to reproduce all the analyses in this paper is available on the Open Science Framework at https://osf.io/wc7zy/ and can also be viewed at https://github.com/sfavila/Favila_NatComm_2022.

For manuscripts utilizing custom algorithms or software that are central to the research but not yet described in published literature, software must be made available to editors and reviewers. We strongly encourage code deposition in a community repository (e.g. GitHub). See the Nature Portfolio guidelines for submitting code & software for further information.

Data

Policy information about availability of data

All manuscripts must include a data availability statement. This statement should provide the following information, where applicable:

- Accession codes, unique identifiers, or web links for publicly available datasets
- A description of any restrictions on data availability
- For clinical datasets or third party data, please ensure that the statement adheres to our policy

Preprocessed MRI data, BOLD activation maps, regions of interest, and behavioral data are deposited on the Open Science Framework at https://osf.io/wc7zy/. Source data are provided with this paper.

Human research participants

Policy information about studies involving human research participants and Sex and Gender in Research.

Reporting on sex and gender	Biological sex was self-reported by participants. Gender was not requested or reported. Sex and gender were not included as covariates in the analyses.
Population characteristics	Human subjects included 5 males and 4 females. Subjects were 22–46 years old at the time of participation. All subjects had normal or corrected-to-normal visual acuity, normal color vision, and no MRI contraindications.
Recruitment	Participants were recruited from the NYU community through flyers posted on campus. Because flyers were posted on a university campus with a large number of undergraduates, participants that volunteered for our study were younger, more educated, and were more comfortable with computer-based tasks than the broader young adult population. This enabled us to have shorter behavioral training sessions to reach high levels of memory performance. We might expect behavioral training to take longer in a young adult population without these characteristics, however we do not expect the main experimental results to differ.
Ethics oversight	New York University Institutional Review Board

Note that full information on the approval of the study protocol must also be provided in the manuscript.

Field-specific reporting

Please select the one below that is the best fit for your research. If you are not sure, read the appropriate sections before making your selection.

☒ Life sciences ☐ Behavioural & social sciences ☐ Ecological, evolutionary & environmental sciences

For a reference copy of the document with all sections, see nature.com/documents/nr-reporting-summary-flat.pdf

Life sciences study design

All studies must disclose on these points even when the disclosure is negative.

Sample size	The number of subjects was chosen based on the observation that previous studies using pRF models in human subjects have produced robust results with 5-10 subjects (Kay et al. Journal of Neurophysiology 2013; Mackey et al. eLife 2017; Benson & Winawer eLife 2018). The number of trials per stimulus for each subject was based on the observation that 40 trials was sufficient to observe reliable perception and memory signals in pilot subjects.
Data exclusions	No subjects or trials were excluded from analysis.
Replication	A replication experiment was not performed in this study.
Randomization	Subjects were not assigned to groups. Within each subject, the following variables were randomized: the assignment of stimuli to locations, the assignment of stimuli to cues, the test probe on each trial during behavioral training, and the order of trials during training and the scan session.
Blinding	Blinding was not relevant because all manipulations were within-subject.

Reporting for specific materials, systems and methods

We require information from authors about some types of materials, experimental systems and methods used in many studies. Here, indicate whether each material, system or method listed is relevant to your study. If you are not sure if a list item applies to your research, read the appropriate section before selecting a response.

Materials & experimental systems

n/a	Involved in the study
☒	☐ Antibodies
☒	☐ Eukaryotic cell lines
☒	☐ Palaeontology and archaeology
☒	☐ Animals and other organisms
☒	☐ Clinical data
☒	☐ Dual use research of concern

Methods

n/a	Involved in the study
☒	☐ ChIP-seq
☒	☐ Flow cytometry
☐	☒ MRI-based neuroimaging

Magnetic resonance imaging

Experimental design

Design type: Event-related task

Design specifications: While being scanned, subjects completed 5--6 runs each of the perception and memory tasks in an interleaved order. This amounted to 40 or 48 trials per stimulus per subject in each task. For both perception and memory runs, trials were 3 seconds long and separated by a fixation period of 3, 4, 5, or 6 seconds.

In a separate session, subjects completed 6 or 12 runs of a retinotopy task. Each run contained 8 full-field or half-field sweeps of a bar across the visual field and took 192 seconds.

Behavioral performance measures: Button presses and response times were recorded for both the main perception and memory tasks and for the retinotopy session. Mean accuracy and range of accuracy across subjects was used to evaluate performance.

Acquisition

Imaging type(s): functional and structural

Field strength: 3 Tesla

Sequence & imaging parameters: Functional images were acquired with a T2*-weighted multiband gradient echo EPI sequence with whole-brain coverage (repetition time = 1 s, echo time = 37 ms, flip angle = 68 degrees, 66 slices, 2 x 2 x 2 mm voxels, multiband acceleration factor = 6, phase-encoding = posterior-anterior). Slices were aligned parallel to the anterior commissure– posterior commissure axis. Spin echo images with anterior-posterior and posterior-anterior phase-encoding were collected to estimate the susceptibility-induced distortion present in the functional EPIs. Between one and three whole-brain T1-weighted MPRAGE 3D anatomical volumes (.8 x .8 x .8 mm voxels) were also acquired for seven subjects. For two subjects, previously acquired MPRAGE volumes (1 x 1 x 1 mm voxels) from a different scanner were used.

Area of acquisition: whole brain

Diffusion MRI ☐ Used ☒ Not used

Preprocessing

Preprocessing software: Anatomical and functional images were preprocessed using FSL and Freesurfer tools implemented in a Nipype workflow. To correct for head motion, each functional image acquired in a session was realigned to a single band reference image and then registered to the spin echo distortion scan acquired with the same phase encoding direction. The two spin echo images with reversed phase encoding were used to estimate the susceptibility-induced distortion present in the EPIs. For each EPI volume, this nonlinear unwarping function was concatenated with the previous spatial registrations and applied with a single interpolation. No slice time correction or spatial smoothing was performed. Freesurfer was used to perform segmentation and cortical surface reconstruction on each subject’s average anatomical volume. Registration from the functional images to each subject's anatomical volume was performed using boundary-based registration. Preprocessed functional time series were then projected onto each subject's reconstructed cortical surface.

Normalization: Data were not normalized. Because this experiment required precise mapping of population receptive fields, which vary in location and size across individuals, all data were kept in native subject space.

Normalization template: Data were not normalized.

Noise and artifact removal	We used a GLM estimation procedure that simultaneously removes noise and artifacts: GLMdenoise. GLMdenoise improves signal-to-noise ratios in GLM analyses by identifying a pool of noise voxels whose responses are unrelated to the task and regressing them out of the time series. This technique first converts all time series to percent signal change and determines an optimal hemodynamic response function for all vertices using an iterative linear fitting procedure. It then identifies noise vertices as vertices with negative R^2 values in the task-based model. Then, it derives noise regressors from the noise pool time series using principal components analysis and iteratively projects them out of the time series of all vertices, one noise regressor at a time. The optimal number of noise regressors is determined based on cross-validated R^2 improvement for the task-based model. This procedure subsumes and outperforms explicitly regressing out motion parameters and physiological recordings from the data.
Volume censoring	No volume censoring was performed.

Statistical modeling & inference

Model type and settings

Mass univariate GLM: We constructed first-level models for each subject, separately for the perception and memory tasks. Design matrices for the perception task were constructed to have four regressors of interest (one per stimulus), with events modeled as boxcars with duration equal to stimulus presentation. Design matrices for the memory task were constructed the same way (one regressor per stimulus), with events modeled as boxcars with duration equal to the cued retrieval period. Prior to estimating the GLM, two separate hemodynamic response functions were estimated for the perception and memory tasks. Hemodynamic response functions were estimated using data from visual regions of interest only. GLM models were run in each subject with a denoising procedure (see noise and artifact removal). These models returned parameter estimates reflecting the BOLD amplitude evoked by perceiving or remembering a given stimulus versus baseline for every vertex on a subject's cortical surface. No group-level modeling was performed.

Encoding model (population receptive field model): Images from the retinotopic mapping session were preprocessed as above, but omitting the final step of projecting the time series to the cortical surface. Using these time series, nonlinear symmetric 2D Gaussian population receptive field (pRF) models were estimated in Vistasoft. Briefly, we estimated the receptive field parameters that, when applied to the drifting bar stimulus images, minimized the difference between the observed and predicted BOLD time series. First, stimulus images were converted to contrast apertures and downsampled to 101 x 101 grids. Time series from each retinotopy run were resampled to anatomical space and restricted to gray matter voxels. Time series were then averaged across runs. pRF models were solved using a two stage coarse-to-fine fit on the average time series. The first stage of the model fit was a coarse grid fit, which was used to find an approximate solution robust to local minima. This stage was solved on a volume-based time series that was first temporally decimated, spatially blurred on the cortical surface, and spatially subsampled. The parameters obtained with this fit were interpolated and then used as a seed for subsequent nonlinear optimization, or fine fit. This procedure yielded four final parameters of interest for every voxel: eccentricity (r), polar angle (theta), sigma (sigma), exponent (n). Variance explained by the pRF model with these parameters was also calculated for each voxel. All parameters were then projected from each subject's anatomical volume to the cortical surface.

Effect(s) tested

Inference was not performed directly on either the GLM or pRF parameters alone. Instead, these parameters were combined to assess population spatial tuning properties. We assessed spatial tuning by selecting the (x, y) parameters for each surface vertex from the retinotopy model and the beta parameters from the GLM analysis. We restricted our analysis to surface vertices whose (x,y) coordinates were within one sigma of the stimulus eccentricity for each ROI. We then binned the beta parameters into 18 bins of polar angle distance from the stimulus and averaged within each bin to produce polar angle response functions for each subject. After combining the data across subjects, we fit a difference of two von Mises distributions to the average data, with the location parameters (mu) for the two von Mises distributions fixed to be equal, but the spread (kappa) and scale allowed to differ. We quantitatively assessed the similarities and differences between perception and memory responses using these fits. We examined the location parameter of the two von Mises distributions, and also computed the amplitude and FWHM of the fit. We repeated the fitting procedure 500 times, drawing subjects with replacement, to create bootstrapped 68% and 95% confidence intervals for both perception and memory location, amplitude, and FWHM parameters. To assess main effects of ROI, main effects of perception vs memory, and the interaction of these variables on location, amplitude, and FWHM values, we ran two-way ANOVAs. We re-ran the ANOVAs for all 500 subject resamplings to create bootstrapped confidence intervals for ANOVA regression coefficients. We computed two-tailed p-values for these effects by performing randomization tests.

Specify type of analysis: ☐ Whole brain ☒ ROI-based ☐ Both

Anatomical location(s): Visual areas V1, V2, V3, hV4, LO (LO1 and LO2), and V3ab (V3a and V3b) were defined by hand for each subject. Definitions were guided by anatomical landmarks and polar angle and eccentricity parameters derived from each subject's retinotopy scan.

Statistic type for inference (See Eklund et al. 2016)	No inference was made directly on voxel-wise maps, but voxel-wise parameters were carried forward into spatial tuning analyses (described above)
Correction	No inference was made directly on voxel-wise maps, so no correction was performed.

Models & analysis

n/a	Involved in the study
☒	☐ Functional and/or effective connectivity
☒	☐ Graph analysis
☐	☒ Multivariate modeling or predictive analysis

Multivariate modeling and predictive analysis	We used our pRF model (see description of fit in statistical modeling section) to generate predicted responses to our experimental stimuli. We first transformed our experimental stimuli into binary contrast apertures with values of 1 where the stimulus was and values of 0 everywhere else and then downsampled these images to the same resolution as the images used to fit the pRF model (101 x 101). We then applied the model to these inputs. After generating a predicted BOLD response for each subject, stimulus, and surface vertex, we carried these predictions forward through the same analysis pipeline used to analyze our actual data. We correlated the predicted location, amplitude, and FWHM parameters for each ROI with the actual perception and memory parameters. We evaluated these relationships by fitting a linear model to the predicted versus observed observations. To generate confidence intervals on these fits, we fit linear models to the 500 bootstrapped perception and memory datasets and the yoked pRF predictions. We also computed the accuracy of the model predictions. We calculated the coefficient of determination R^2 for the predicted polar angle response functions in each ROI, separately for the observed perception and memory polar angle response functions. We generated confidence intervals for these accuracies by computing R^2 values for each of the 500 bootstrapped perception and memory datasets and the yoked pRF predictions.
